# Electrospun nano-fibrous bilayer scaffold prepared from polycaprolactone/gelatin and bioactive glass for bone tissue engineering

**DOI:** 10.1007/s10856-021-06588-6

**Published:** 2021-08-28

**Authors:** Hend Elkhouly, Wael Mamdouh, Dalia I. El-Korashy

**Affiliations:** 1 Biomaterials Department, Faculty of Dentistry, Ain Shams University, Organization of African Unity St., El-Qobba Bridge, Al Waili, Cairo, 11566 Egypt; 2 Department of Chemistry, School of Sciences and Engineering, The American University in Cairo (AUC), AUC Avenue, P.O. Box 74, New Cairo, 11835 Egypt

## Abstract

This work is focused on integrating nanotechnology with bone tissue engineering (BTE) to fabricate a bilayer scaffold with enhanced biological, physical and mechanical properties, using polycaprolactone (PCL) and gelatin (Gt) as the base nanofibrous layer, followed by the deposition of a bioactive glass (BG) nanofibrous layer via the electrospinning technique. Electrospun scaffolds were characterized using scanning electron microscopy (SEM), transmission electron microscopy (TEM) and Fourier transform infrared spectroscopy. Surface area and porosity were evaluated using the nitrogen adsorption method and mercury intrusion porosimetry. Moreover, scaffold swelling rate, degradation rate and in vitro bioactivity were examined in simulated body fluid (SBF) for up to 14 days. Mechanical properties of the prepared scaffolds were evaluated. Cell cytotoxicity was assessed using MRC-5 cells. Analyses showed successful formation of bead-free uniform fibers and the incorporation of BG nanoparticles within fibers. The bilayer scaffold showed enhanced surface area and total pore volume in comparison to the composite single layer scaffold. Moreover, a hydroxyapatite-like layer with a Ca/P molar ratio of 1.4 was formed after 14 days of immersion in SBF. Furthermore, its swelling and degradation rates were significantly higher than those of pure PCL scaffold. The bilayer’s tensile strength was four times higher than that of PCL/Gt scaffold with greatly enhanced elongation. Cytotoxicity test revealed the bilayer’s biocompatibility. Overall analyses showed that the incorporation of BG within a bilayer scaffold enhances the scaffold’s properties in comparison to those of a composite single layer scaffold, and offers potential avenues for development in the field of BTE.

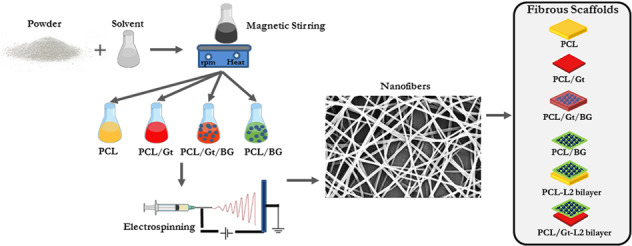

## Introduction

One of the main concerns that precede the placement of dental implants is an inadequacy of bone tissue at the implant site [1]. In fact, severe periodontal diseases, cancer, trauma, infections, as well as other degenerative diseases, all result in bone defects and bone loss [2]. The quality of life of such patients is affected as a result of pain, disfigurement or loss of function. Accordingly, different types of bone grafts have been used as filling materials to replace lost bone. For instance, autogenous bone grafts have shown good clinical outcomes, decreasing the risk of rejection that is commonly seen when using allografts; however, the ability to harvest large amounts of bone from patients is limited [3].

To overcome the drawbacks of traditional bone grafting materials, bone tissue engineering (BTE) was introduced, offering biodegradable scaffolds which could be fabricated from a wide variety of materials such as polymers, bio-ceramics, metals, etc. These scaffolds act as supporting structures for cell adhesion, infiltration, proliferation and differentiation [1].

Various fabrication methods have been pursued for scaffolds [4–7], including nascent techniques from nanotechnology [8–10]. In particular, electrospinning is one of the most widely used techniques to fabricate fibrous membranes and scaffolds. It is a versatile method which allows the use of many different polymers and inorganic materials as well as the integration of drugs, signaling molecules and cells. Fiber diameters range from the micro-scale down to the nano-scale, yielding a high surface area which is favorable for cell adhesion and proliferation. One of the most important advantages of using electrospinning to produce fibrous scaffolds is the control it offers over their uniformity (size, length, arrangement, etc.). Normally, scaffolds produced by electrospinning are highly porous and have an inter-connected pore structure mimicking the physical structure of the extracellular matrix (ECM) [11, 12].

There are many types of synthetic polymers used to prepare fibrous scaffolds by electrospinning and were proven successful in BTE, one of which is polycaprolactone (PCL) which is a biodegradable polyester [4, 13]. It has many advantages such as its biocompatibility, non-toxic degradation products, easy handling and good mechanical properties. However, its hydrophobic nature limits cellular response in addition to its slow degradation rate [14]. To improve the scaffold’s properties, blending PCL with natural polymers such as collagen, chitosan and gelatin (Gt) has been widely reported [11, 14–17].

Gt is a natural polymer which has many advantages such as its hydrophilicity, biocompatibility, faster degradation rate and its availability at relatively low cost, all of which have contributed to its wide use in the field of BTE. However, its main disadvantage is that it lacks bioactivity [11]. Pereira, et al. [16] showed that there was osteogenic differentiation and proliferation from human adipose stem cells on mineralized PCL/Gt mats, enabling their use in BTE. In addition, Duan, et al. [18] tested the biocompatibility of Gt/PCL nanofibers and showed that the blends allowed human cultured keratinocytes to adhere and spread on the scaffolds after 24 h of seeding.

Bioactive glasses (BG) are a group of surface-reactive biocompatible glass-ceramics. They have gained potential interest in the BTE field as they form a hydroxyapatite-like layer when in close contact with the host tissue. Research has proven that close contact between this layer and the ECM stimulates osteoblasts to regenerate bone [19]. Liverani, et al. [20] used electrospinning to incorporate BG nanoparticles into PCL/chitosan nanofibers and their results showed that the BG nanoparticles preserved their bioactivity as hydroxyl-carbonated apatite precipitates began to form after one day of immersion in simulated body fluid (SBF). Moreover, Shirani, et al. [21] blended PCL with Gt and BG in one composite scaffold using electrospinning and their results showed that adding BG nanoparticles enhanced the scaffold’s mechanical properties as well as its cellular response.

The novelty in this study is the incorporation of PCL, Gt and BG into an electrospun bilayer scaffold. This was achieved by blending PCL with Gt to form a nanofibrous base layer, and surface coating it with a layer of BG nanofibers. We then compare its properties to those of a composite single layer scaffold.

## Materials and methods

### Solution preparation

The PCL solution was prepared by dissolving 14% (w/v) of PCL pellets (average Mw 80,000, Sigma Aldrich, USA) in formic acid (98–100%, Fisher Chemical, Fisher Scientific, Sweden), and was left to stir overnight on a magnetic stirrer at 400 rpm. The PCL/Gt solution was prepared by dissolving and stirring 14% PCL in formic acid for 3 h until a clear solution was obtained followed by the addition of 30 w% (with respect to PCL weight) of Gt from porcine skin (gel strength ~300 g Bloom, Type A, Sigma Aldrich, USA), and was left to stir overnight at 400 rpm.

The BG nanoparticles (Bioglass nanoparticles; Nanostreams, Egypt: NS0001) were manufactured by the sol-gel technique with a composition of 45% silica, 25% CaO, 25% Na_2_O, and 5% P_2_O_5,_ and a particle size of less than 10 nm. The PCL/Gt/BG composite scaffold was prepared by adding 3 w% (with respect to PCL weight) of BG to a PCL/Gt solution prepared as previously mentioned, where 0.042 g BG powder was weighed and dispersed in the PCL/Gt solution after complete dissolution of the polymers. The composite solution was left to stir overnight at 600 rpm and then was placed in an ultrasonic bath for 10 min and that solution was immediately electrospun [20]. On the other hand, to obtain BG nanofibers and due to the difficulty of spinning BG alone, PCL was chosen as a suitable polymer to incorporate BG. The PCL/BG solution was prepared by dissolving 14% PCL in formic acid for 3 h, after which 3% BG powder was added and left to stir overnight at 600 rpm, then was placed in an ultrasonic bath for 10 min and immediately electrospun. The preparation of solutions was done at room temperature (22 ± 1 °C).

### Electrospinning process

An in-house electrospinner was used for the electrospinning process. It was composed of a syringe pump (Shenzhen Shenke Medical Instrument Technical Development, China), a high voltage power supply (Gamma, USA), 10 mL plastic syringes, silicone rubber tubes and a stationary copper plate collector covered with an aluminum foil sheet. A plastic syringe was filled with 10 mL of each prepared solution and was attached to the syringe pump. The silicone rubber tube was used to connect the tip of the syringe to a 21 gauge metallic needle of diameter 0.82 mm. An electric circuit was achieved by connecting the needle tip to the high voltage power supply. Upon adjusting the electrospinning parameters (solution flow rate, applied voltage and the distance between the spinneret tip and the collector), fibers were collected onto an aluminum foil sheet, which was wrapped around a copper metal plate collector. The PCL, PCL/Gt/BG and PCL/BG solutions were electrospun at a flow rate of 0.5 mL/h and a voltage of 17 kV. The PCL/Gt solution was electrospun at a flow rate of 1.0 mL/h using a voltage of 25 kV. The distance between the tip of the needle and the plate collector was kept constant at 13 cm for all experiments [17].

### Bilayer preparation

The bilayer scaffold was prepared by electrospinning PCL/BG nanofibers (L2) directly on top of PCL/Gt electrospun nanofibers to obtain a PCL/Gt-L2 bilayer scaffold. For the purpose of comparison, an additional bilayer was fabricated by electrospinning L2 directly on top of pure PCL nanofibers to obtain a PCL-L2 bilayer scaffold. Solutions of equal volumes (50:50) were used to electrospin both layers.

### Characterization

#### Viscosity of solutions

The viscosity of the different solutions was measured at room temperature (22 °C) using a rotational viscometer (MYR VR 3000, Viscotech, Spain). The solutions were measured at a rotational speed of 5 rpm.

#### Fiber diameter and morphology examination

Field Emission Scanning Electron Microscope (FESM, Leo Supra 55-Zeiss Inc., Germany) was used to evaluate the surface morphology of the fibers and their diameters. Fibers were collected for 5 min for each scaffold. Then, the samples were gold sputtered at 15 mA for 1 min. At ×10,000 magnification, the diameters of 30 fibers from each sample were measured using Image J analysis software [20, 22, 23], and the results were plotted as histograms.

Transmission electron microscope (TEM) (Jeol, JEM 2100, Japan) was used to compare the distribution, location and size of the BG nanoparticles within the nanofibrous scaffolds. Samples for TEM imaging were prepared by depositing electrospun fibers onto copper grids for 10 s to insure the deposition of only a few fibers onto the grid [24].

#### Chemical analysis

Fourier transform infrared (FT-IR) spectroscopic analysis (Nicolet 380-Thermo Scientific, USA) was used to detect the chemical characteristics of all scaffolds in the transmittance mode with mid-IR region of (4000–500 cm^−1^). The fibrous samples were collected and cut into squares of 2 × 2 cm^2^ for FTIR analysis.

#### Surface area and porosity tests

The surface area of the prepared scaffolds was determined using nitrogen gas (N_2_) sorption analysis using ASAP 2020 analyzer (Micromeritics Instrument Corporation, Norcross, GA, USA). Samples were prepared by electrospinning 12 mL of the different solutions according to the previously mentioned parameters. As for the bilayers, each layer was collected from a 6 mL solution. The collected fibrous sheets were cut into squares of 5 × 5 mm^2^ and ~0.5–0.7 g of these fibers were used. For Brunauer, Emmett, and Teller (BET) analysis, the samples were degassed at 40 °C for 4 h under a vacuum of 0.01 mmHg. The adsorption and desorption isotherms were recorded using 48-point pressure tables with 20-s equilibrium intervals. BET was used to calculate the surface area using a model of adsorption involving multilayer coverage. To calculate the size distributions of meso and macropores, the method of Barrett, Joyner and Halenda (BJH) was carried out using the Kelvin model of pore filling. Moreover, mercury intrusion porosimetry (Pore sizer; model 9320, Micrometrics USA) was used to measure the mean pore diameter for each sample as well as their pore volume. Mercury was forced into the pores using pressures up to 30,000 Psi. Samples were degassed at 50 µmHg evacuation pressure for 30 min and ~0.05–0.1 g of the samples were used.

#### In vitro bioactivity

Samples used for in vitro bioactivity testing were prepared by cutting the fibrous scaffolds into squares of dimensions 1 × 1 cm^2^ and immersing them in polyethylene tubes filled with 30 mL of freshly prepared SBF, which was prepared according to the protocol suggested by Kokubo et al. [25]. To avoid confusion during SEM imaging, the bilayers were distinguished by drawing a small blue dot on L2 using a blue ink pen. Samples were removed at 2, 4, 7, and 14 days of immersion. Five samples from each scaffold were tested for each time interval (*n* = 20). The amount of SBF was added according to the following formula [25]:

1 $${{{{{\mathrm{V}}}}}} = {{{{{\mathrm{SA}}}}}}/10$$

where, V is the volume of SBF in mL and SA is the surface area of the film in mm^2^. All samples were left in a shaking incubator at 37 °C and at a shaking speed of 90 rpm [17]. SBF was refreshed every 48 h. The samples were removed after each of the specified immersion periods, washed thoroughly with distilled water, then patted using filter paper and dried in the incubator at 37 °C until a constant mass was obtained [2]. The samples were then gold sputtered and examined using SEM, coupled with energy dispersive x-ray analysis (EDX) for elemental analysis [26, 27].

#### Cell cytotoxicity using MTT assay

The MTT assay was used to test the cytotoxicity of the PCL/Gt-L2 nanofibrous bilayer scaffold. The samples were cut into 1 × 1 cm^2^ and sterilized using UV light for 2 h on each side (*n* = 3). MRC-5 cells (normal human lung fibroblasts) obtained from the American Type Culture Collection (ATCC, Rockville, MD) were used after 24 h of seeding. The microtiter plates were incubated at 37 °C in a humidified incubator with 5% CO_2_ for a period of 48 h. Three wells were used for the test sample. Control cells were incubated without the test sample. After incubating for 48 h, the numbers of viable cells were determined by the MTT test. Briefly, the media was removed from the 6 well plate and replaced with fresh culture RPMI 1640 medium without phenol red then 100 µl of the 12 mM MTT stock solution (5 mg of MTT in 1 mL of PBS) to each well including the untreated controls. The 6 well plates were then incubated at 37 °C under 5% CO_2_ for 4 h. An 850 µl aliquot of the media was removed from the wells, and 500 µl of DMSO was added to each well and mixed thoroughly with the pipette and incubated at 37 °C for 10 min. Then, the optical density was measured at 590 nm with the microplate reader (SunRise, TECAN, Inc, USA) to determine the number of viable cells and the percentage of viability was calculated as follows:

2 $$({{{{{\mathrm{ODt}}}}}}/{{{{{\mathrm{ODc}}}}}}){{{{{\mathrm{x}}}}}}100\%$$

where, ODt is the mean optical density of wells treated with the tested sample and ODc is the mean optical density of untreated cells. Results were recorded as means and standard deviation, followed by statistical analysis using one-way *t* test.

#### Evaluation of scaffold swelling and degradation rates

To evaluate the swelling rates of different scaffolds, the initial weights of the samples were measured before immersion in SBF. In this test, five samples were tested for each scaffold at each time interval (*n* = 15). Then, the samples were removed from the SBF after 2, 7, and 14 days, washed with distilled water, and then were dried using filter paper to remove any excess water [28]. The samples were then weighed and the swelling ratio (S) was calculated using the following formula [28]:

3 $${{{{{\mathrm{S}}}}}}(\% ) = ({{{{{\mathrm{WS}}}}}} - {{{{{\mathrm{W}}}}}}0)/{{{{{\mathrm{W}}}}}}0{{{{{\mathrm{X}}}}}}100$$

where, W_0_ is the weight of the sample (in grams) before immersion in SBF and W_S_ is the weight of the swollen sample (in grams).

As for degradation rate test, after the removal of the samples from SBF, they were washed with distilled water, then patted using filter paper and dried in an incubator until a constant mass was obtained. The samples’ weights were measured after complete dryness [23]. Weight loss (WL) was calculated using the following formula [29]:

4 $${{{{{\mathrm{WL}}}}}}({{{{{\mathrm{\% }}}}}}) = ({{{{{\mathrm{W}}}}}}0 - {{{{{\mathrm{WD}}}}}})/{{{{{\mathrm{W}}}}}}0\,{{{{{\mathrm{X}}}}}}\,100$$

where, W_0_ is the weight (in grams) of the sample before immersion and W_D_ is the weight (in grams) of the sample after removal from the SBF and completely dried.

#### Mechanical testing

For the mechanical tests of the fabricated scaffolds, a universal testing machine (Instron 5569 Load frame, USA) with a 5 kN load cell capacity was used to determine the tensile strengths and elastic moduli of all scaffolds. Five samples were tested for each scaffold (*n* = 5). All samples were cut into rectangular films of 50 mm length and 5 mm width and the thicknesses of the samples were measured using a film thickness gauge. The samples were gripped on both sides by adhesive tape and their gauge length was 30 mm each [30]. All samples were pulled using a cross head speed of 1.8 mm/min.

#### Statistical analysis

Results were collected, tabulated and presented as means and standard deviations. Statistical analysis was done using one-way of variance (ANOVA), with a *p* value < 0.05 coupled with Tukey’s multiple comparison tests for pairwise comparisons.

## Results

### Viscosity measurements

The viscosities of the prepared solutions are shown in Table 1. The addition of Gt to PCL led to a 20.2% increase in viscosity, while the addition of BG nanoparticles only led to 5% increase in viscosity. Moreover, the PCL/Gt/BG composite solution was 26.1% more viscous than the PCL solution.

**Table 1:** Viscosities of the prepared samples

Sample	Viscosity (mPas)
PCL	409
PCL/Gt	652
PCL/BG	469
PCL/Gt/BG	723

### Fiber diameter and morphology

**Fig. 1:**
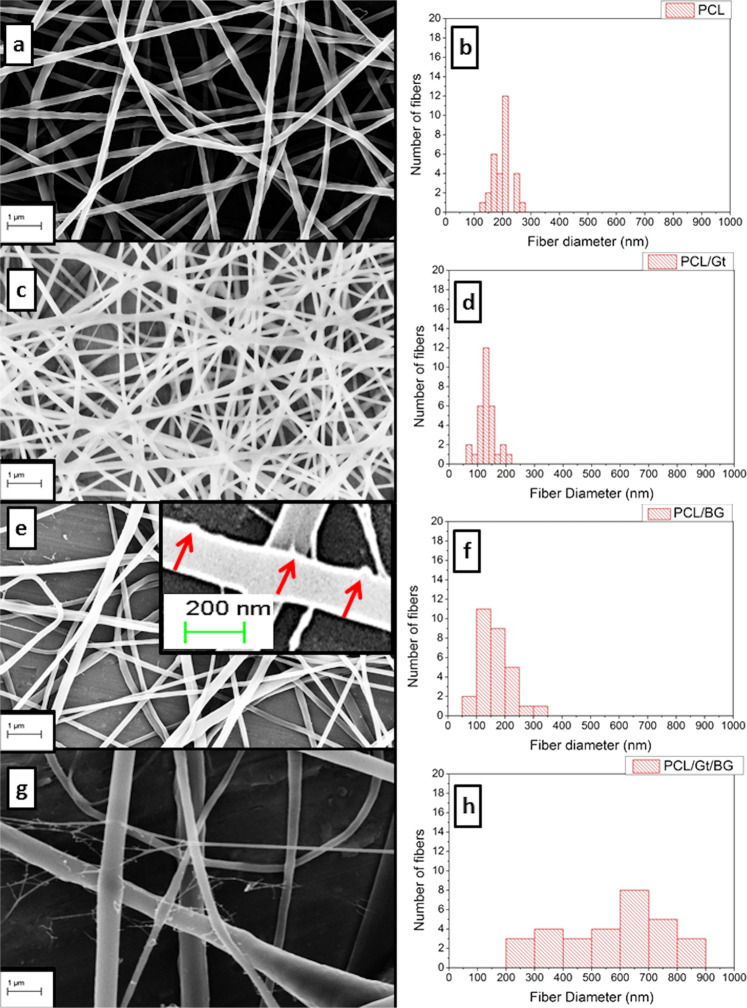
SEM images and their corresponding histograms of the fiber diameters of (**a**, **b**) PCL, (**c**, **d**) PCL/Gt, (**e**, **f**) PCL/BG and (**g**, **h**) PCL/Gt/BG electrospun nanofibers. Arrows indicate areas of surface roughness in PCL/BG fibers

**Fig. 2:**
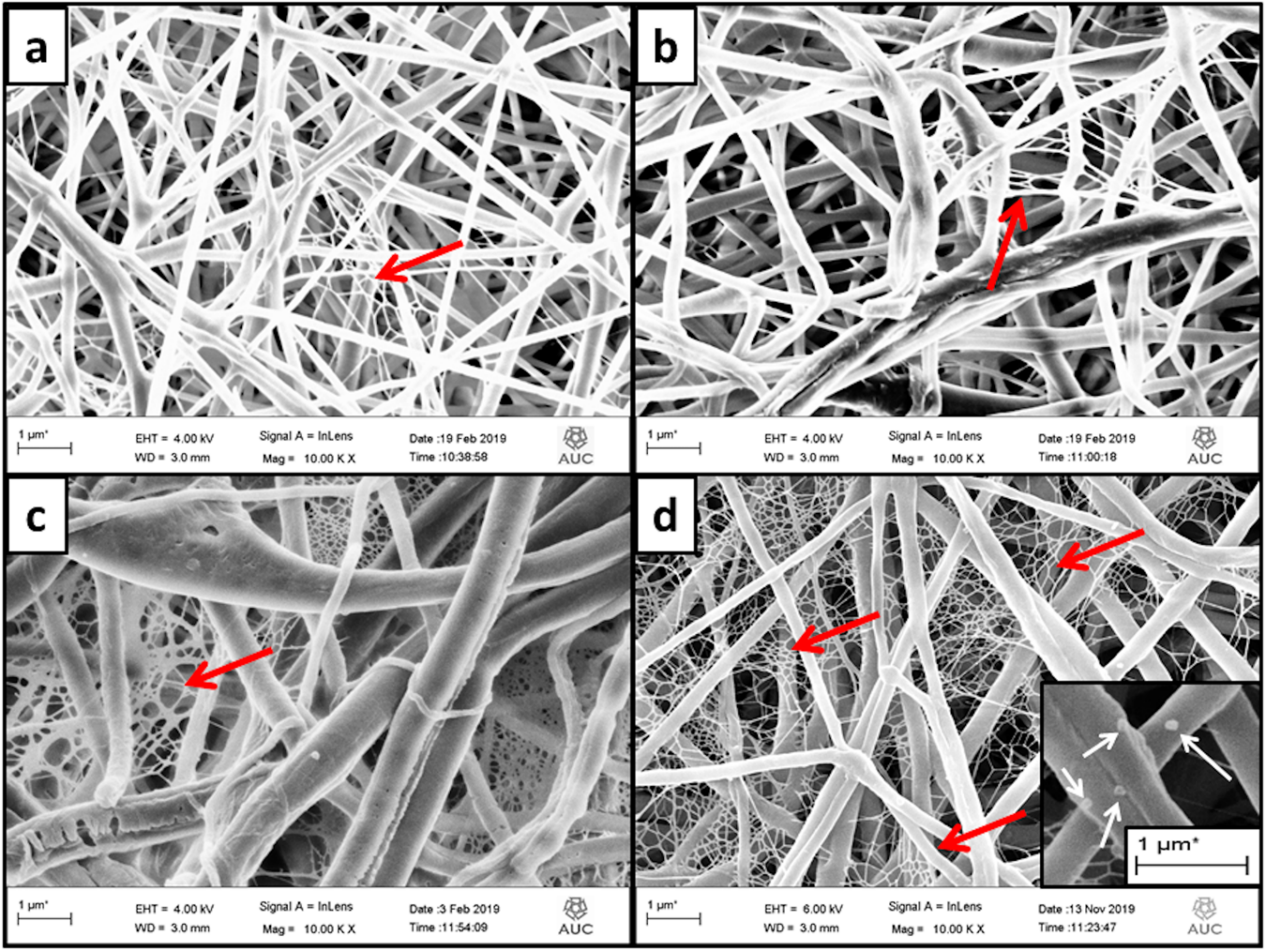
SEM images showing different distributions of nanonets present in (**a**) PCL, (**b**) PCL/Gt, (**c**) PCL/Gt/BG and (**d**) PCL/BG electrospun nanofibers. Arrows indicate nanonets. The inset in (**d**) shows the incorporation of BG nanoparticles within PCL/BG nanofibers as indicated by white arrows

**Fig. 3:**
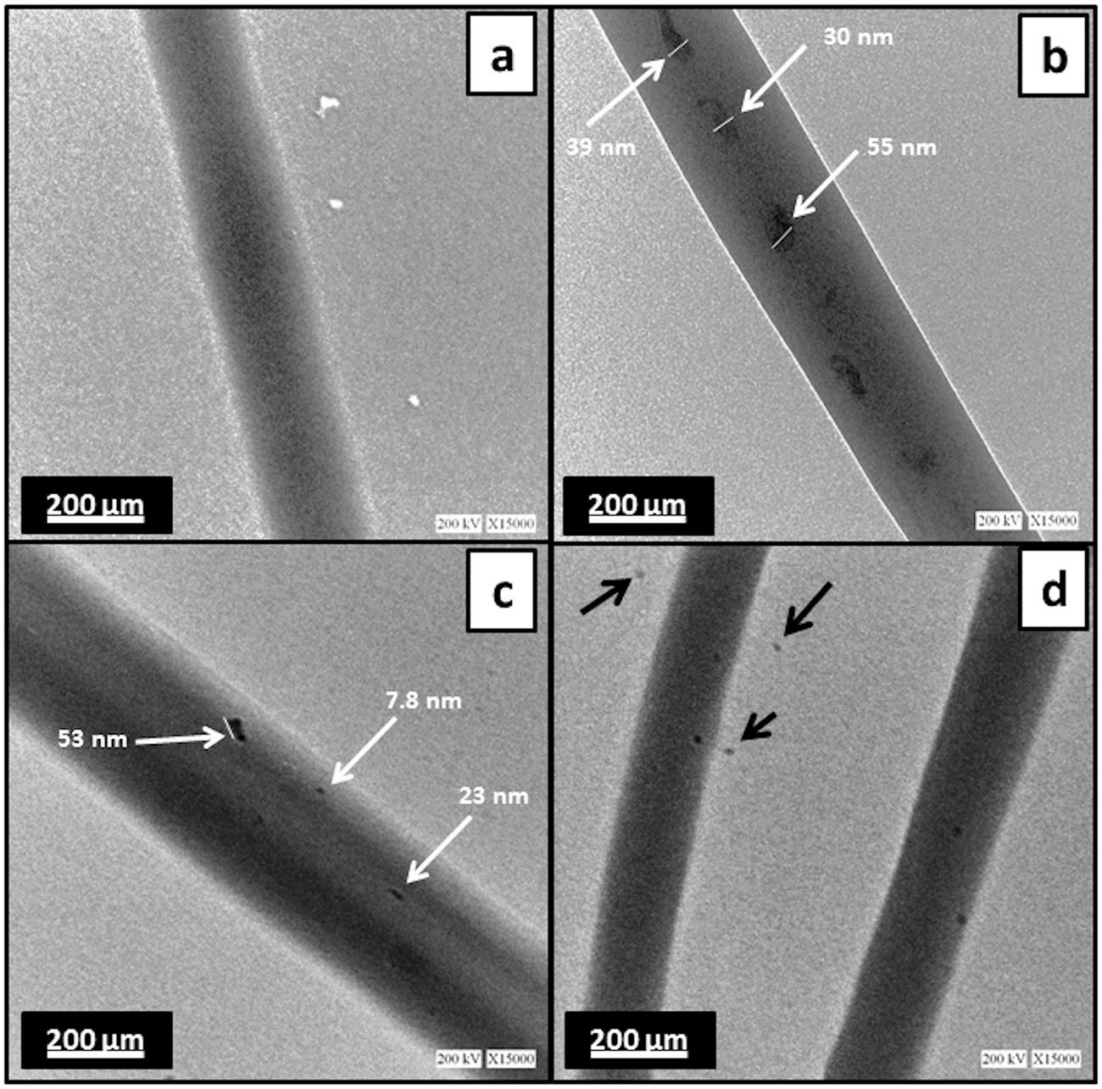
TEM images of (**a**) PCL, (**b**) PCL/BG and (**c**, **d**) PCL/Gt/BG electrospun nanofibers. White arrows indicate nanoparticles within fibers. Black arrows indicate nanoparticles dispersed onto the copper grid

The SEM images for PCL, PCL/Gt, PCL/Gt/BG, and PCL/BG nanofibers and their corresponding histograms are shown in Fig. 1. Uniform bead-free fibers were successfully formed. The uniformity of the electrospun nanofibers was enhanced by the addition of Gt to PCL. Moreover, the morphology of PCL fibers became rough with the addition of BG nanoparticles as shown in Fig. 1e. The fiber diameters of pure PCL nanofibers showed a mean value of 198.69 ± 32.82 nm. However, after incorporating Gt into the PCL solution, the fiber diameter of PCL/Gt nanofibers showed a mean value of 131.80 ± 29.72 nm, which was significantly (*p* = 0.0482) lower than that of pure PCL fibers. Surprisingly, PCL/Gt/BG composite scaffold showed a mean fiber diameter of 565.57 ± 185.50 nm, which was significantly (*p* < 0.0001) higher than all electrospun scaffolds. On the other hand, the mean fiber diameter for the PCL/BG scaffold was 162.09 ± 51.96 nm. Interestingly, some ultrathin fibers (nanonets) ranging between 15 and 30 nm in diameter and pore sizes ranging from 80 to 350 nm were clearly visible in the scaffolds containing BG (Fig. 2). As can be seen in Fig. 3, TEM images revealed the incorporation of nanostructures ranging from 7.8 to 55 nm within the PCL/BG and PCL/Gt/BG nanofibers. However, the PCL/Gt/BG nanofibers contained fewer nanostructures, which were also seen dispersed outside the fibers and onto the grid, as indicated by arrows in Fig. 3d. It is worth remarking that these nanostructures could not be seen in the TEM images of the pure PCL nanofibers (Fig. 3a).

### Chemical analysis

**Fig. 4:**
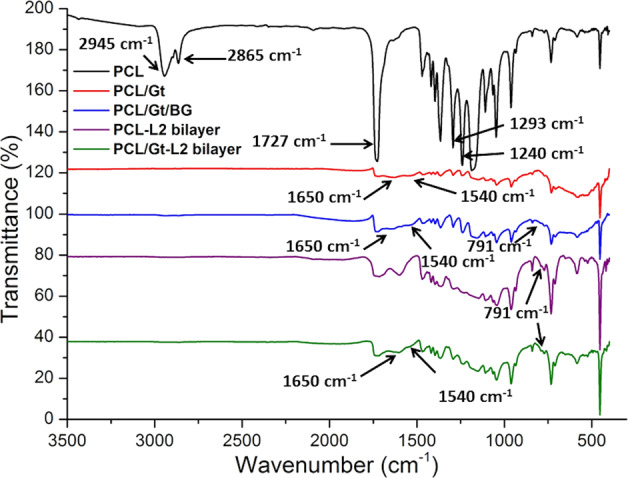
FTIR spectra of the prepared scaffolds in the wavenumber range of 500–3500 cm^−1^

FTIR spectra of the different scaffolds are illustrated in Fig. 4. The common PCL bands observed at 2945 cm^−1^ corresponded to asymmetric CH_2_ stretching, while those observed at 2865 cm^−1^ appeared to be due to symmetric CH_2_ stretching [20, 23]. Moreover, the peaks observed at 1727 cm^−1^ were ascribable to carbonyl stretching, while those observed at 1293 cm^−1^ and 1240 cm^−1^ to C-O, C-C stretching and to asymmetric COC stretching [27]. The only difference between the PCL and PCL/Gt scaffolds was the appearance of a new band at 1540 cm^−1^ which was ascribable to the amide II group (NH_2_) and broadening of the peak at 1650 cm^−1^ (amide I) [23]. These peaks were also found in the PCL/Gt/BG composite scaffold and the PCL/Gt-L2 bilayer spectra. Surprisingly, the introduction of BG nanoparticles gave rise to a new peak observed at 791 cm^−1^ which was most probably due to Si-O-Si bending [20]. This peak was very weak in PCL/Gt/BG composite scaffold. Moreover, broadening of the peak occurred at ~1150–1185 cm^−1^, which was ascribable to Si-O-Si bonds [20].

### Surface area and porosity

The surface area of the prepared scaffolds measured by BET showed that the PCL scaffold had the highest surface area as shown in Table 2. The PCL/Gt-L2 bilayer scaffold had higher surface area than the composite PCL/Gt/BG scaffold by ~15%. Regarding the total pore volume of the prepared scaffolds, the trend shown by mercury intrusion porosimetry coincided with that of the N_2_ adsorption method with the exception of the PCL/Gt scaffold, which showed higher total pore volume than both bilayers using mercury intrusion porosimetry; however, showed less porosity using the N_2_ adsorption method. Interestingly, the total pore volume of the PCL/Gt-L2 bilayer was 6% higher than the PCL/Gt/BG composite scaffold as measured by the mercury intrusion porosimetry.

**Table 2:** Mean pore diameter in (nm), total pore volume (%) measured by mercury intrusion porosimetry, BET surface area and total pore volume measured by N_2_ adsorption method of the prepared scaffolds

Scaffold	Mean pore diameter (nm)	Total pore volume (%)	BET surface area (m^2^/g)	N_2_ adsorption total pore volume (cm^3^/g)
PCL	65.6	49.0	10.10	0.0377
PCL/Gt	104.8	48.4	4.15	0.0100
PCL/Gt/BG	61.7	39.3	3.31	0.0090
PCL-L2 bilayer	74.8	46.5	8.90	0.0274
PCL/Gt-L2 bilayer	95.8	45.6	3.91	0.0108

**Fig. 5:**
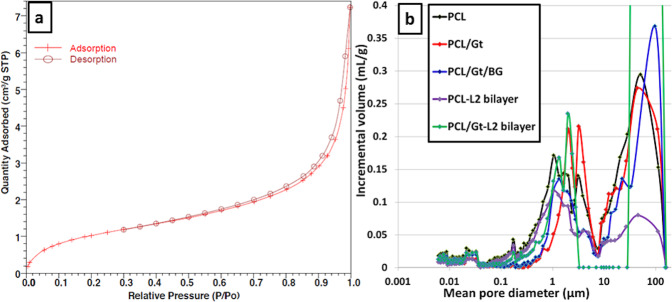
Graphical representation of the (**a**) adsorption-desorption isotherm of PCL/Gt-L2 bilayer and (**b**) pore size distribution measured by the mercury intrusion porosimeter

The adsorption-desorption isotherms produced from the N_2_ adsorption method showed the same pattern for all scaffolds. The curves showed that the scaffolds exhibited a type II isotherm with H3 type hysteresis loop [31, 32]. Figure 5a shows the adsorption-desorption isotherm of a representative scaffold. The mercury intrusion porosimeter revealed that all scaffolds had a wide range of pore diameters from 6 nm up to 100 µm (Fig. 5b). In fact, all scaffolds showed the same trend up to ~0.170 µm, after which they showed a difference in pore size distribution, where the PCL/Gt scaffold and PCL/Gt-L2 bilayer scaffold showed a high volume of pore diameters around 2 µm.

### In vitro bioactivity

**Fig. 6:**
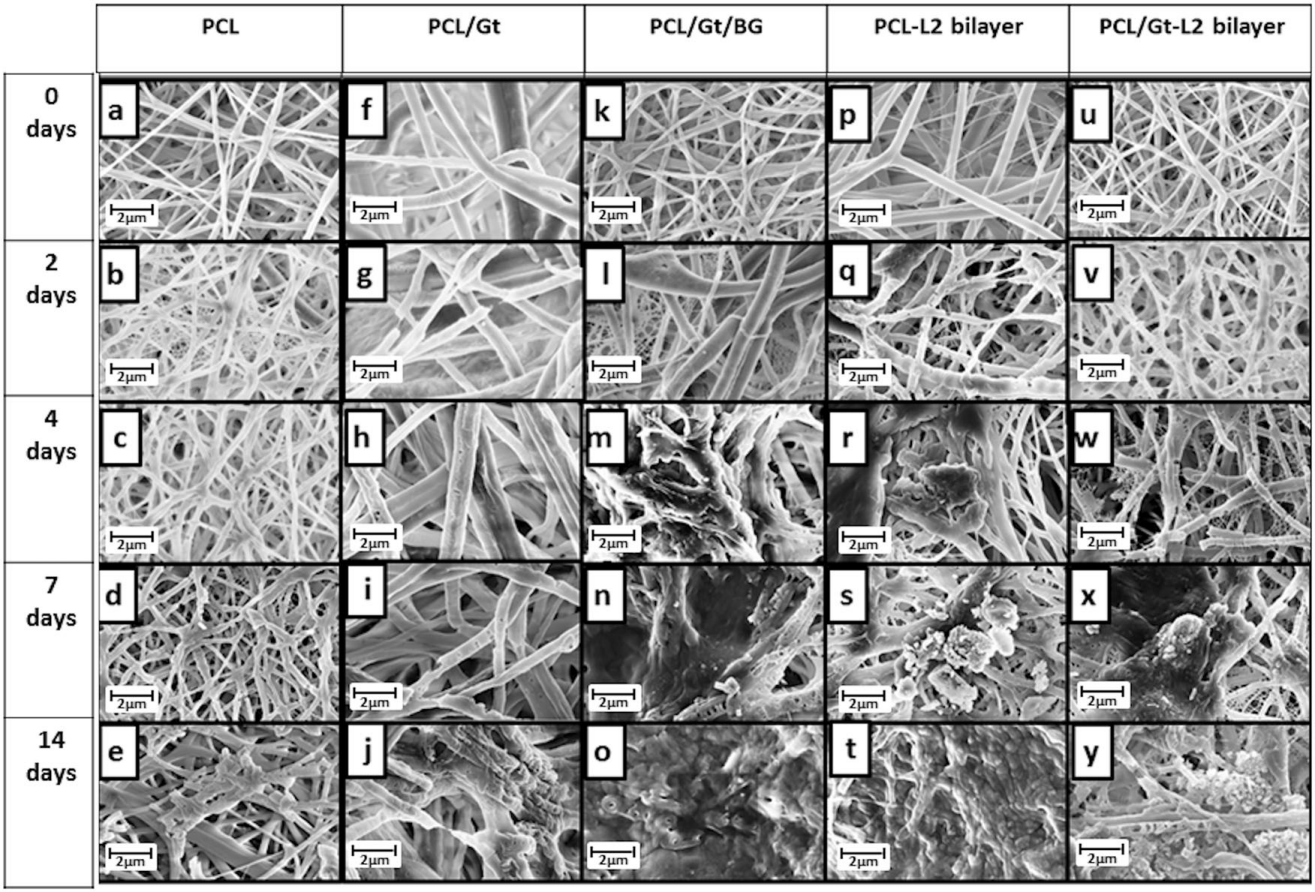
SEM images showing the morphologies of different electrospun scaffolds at 0, 2, 4, 7, and 14 days of immersion in SBF

**Fig. 7:**
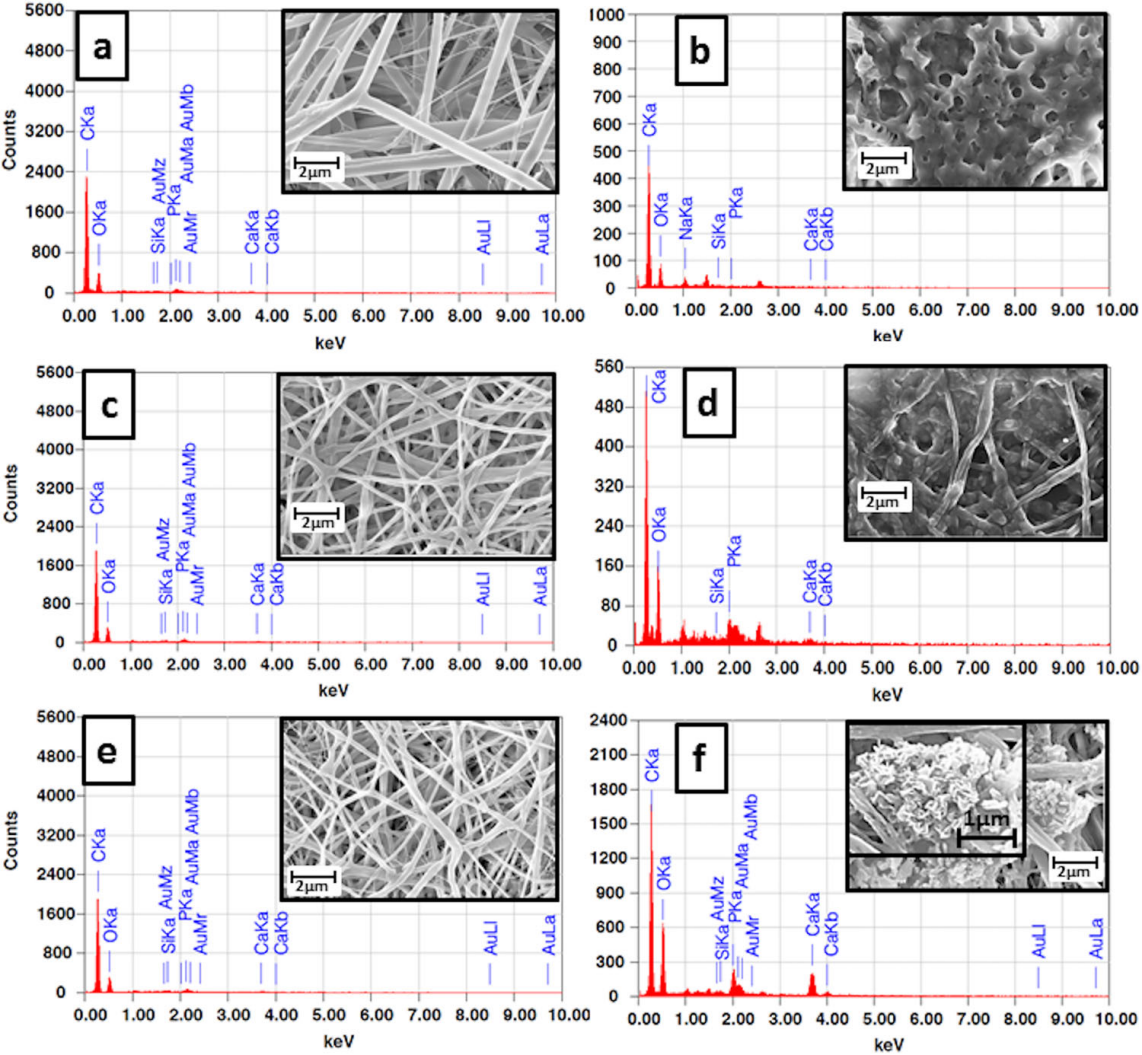
EDX and superimposed SEM images of (**a**, **b**) PCL/Gt/BG composite scaffold, (**c**, **d**) PCL-L2 and (**e**, **f**) PCL/Gt-L2 bilayer scaffold before and after 14 days of immersion in SBF. The inset in (**f**) shows the cauliflower shaped structures which appeared in the PCL/Gt-L2 scaffold after 14 days of immersion in SBF

As can be seen in Fig. 6, the changes in surface morphology observed in the SEM images for PCL and PCL/Gt nanofibers during their period of immersion in SBF were due to the breakdown and scission of the nanofibers as a result of fiber degradation. On the other hand, in addition to signs of degradation, SEM images of PCL/Gt/BG, PCL-L2 bilayer and PCL/Gt-L2 bilayer revealed a deposited layer which began to form with time after their immersion in SBF. By day 14, almost the entire surface of the scaffolds was covered with this deposition. The PCL/Gt/BG scaffold showed the formation of a distorted globular layer and as shown in Fig. 7b, EDX analysis showed very little phosphorus and calcium with a Ca/P molar ratio of 0.32. On the other hand, the SEM image of the PCL-L2 bilayer showed the formation of defined globular structures and as shown in Fig. 7d. EDX analysis of the bilayer showed an increase in the amount of phosphorus and calcium after 14 days of immersion; however, the calculated Ca/P ratio was 0.20. As for the PCL/Gt-L2 bilayer, the SEM image showed an appearance of the typical cauliflower shaped structures after 14 days of immersion (Fig. 7f) and the resulting Ca/P molar ratio was 1.4.

### Cell cytotoxicity using MTT assay

**Fig. 8:**
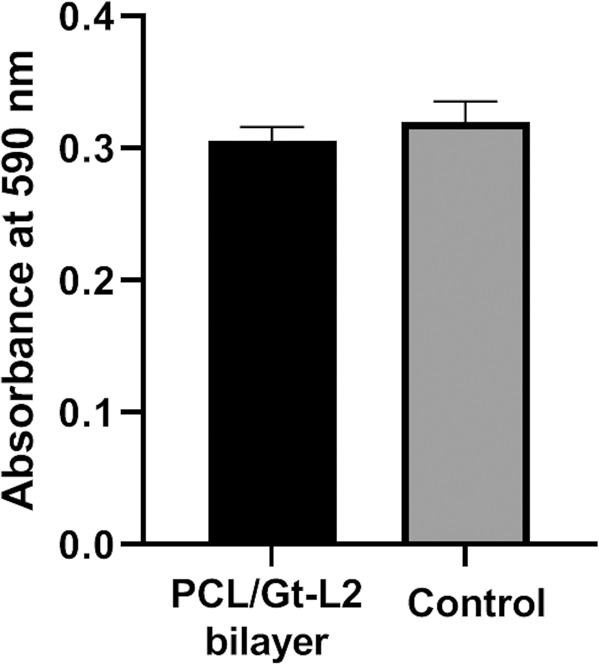
Cell cytotoxicity on PCL/Gt-L2 bilayer and control as measured by optical density using MTT assay. Data are representative of three independent experiments and plotted as mean ± SD (*n* = 3)

The cell cytotoxicity results are shown in Fig. 8. The results revealed that there was no significant difference in the optical density recorded between the PCL/Gt-L2 bilayer and the control (*p* = 0.2582). The mean percentage of cell viability recorded for the bilayer was 92.12% after 48 h of incubation.

### Scaffold swelling and degradation rates

Swelling and degradation tests were carried out to calculate the swelling ratio of the prepared scaffolds after immersion in SBF for 2, 7, and 14 days. Table 3 shows the mean swelling ratios and weight loss percentages for the prepared scaffolds. The PCL scaffold showed the lowest swelling ratio (<15%) amongst all scaffolds throughout the entire duration of immersion. On the other hand, the PCL/Gt and PCL/Gt/BG scaffolds showed the highest swelling ratios at 2 days followed by a significant decrease in swelling at 7 days by ~24% and 35% respectively. On the other hand, the swelling ratio of the PCL-L2 bilayer scaffold significantly increased by 2 and 7 days by fourfold (*p* < 0.0001) and remained to increase till day 14. Finally, the swelling ratio of the PCL/Gt-L2 bilayer scaffold significantly increased by ~28% (*p* = 0.0059) between 2 and 7 days and remained constant thereafter.

**Table 3:** Mean and standard deviation (SD) values of the swelling ratios and weight loss percentages for the prepared scaffolds at 2, 7, and 14 days

Scaffold	Time interval					
	2 days		7 days		14 days	
	Mean swelling ratio ± SD(%)	Mean weight loss ± SD(%)	Mean swelling ratio ± SD(%)	Mean weight loss ± SD(%)	Mean swelling ratio ± SD(%)	Mean weight loss ± SD(%)
PCL	9.462^c^ ± 2.430	0.000 ^b^ ± 0.000	5.944^d^ ± 1.415	2.580^c^ ± 0.3175	12.18^b^ ± 2.554	4.578^c^ ± 0.9768
PCL/Gt	184.3^a^ ± 17.64	15.46^a^ ± 1.192	139.9^ab^ ± 21.19	33.31^a^ ± 4.018	121.9^a^ ± 10.90	31.64^a^ ± 2.668
PCL/Gt/BG	178.2^a^ ± 15.50	14.15^a^ ± 4.722	114.9^bc^ ± 25.75	33.04^a^ ± 5.339	131.9^a^ ± 31.75	31.48^ab^ ± 3.814
PCL-L2 bilayer	23.64^c^ ± 6.737	1.346^b^ ± 0.4999	98.75^c^ ± 18.34	−3.475^c^ ± 1.055	125.0^a^ ± 9.028	−1.993^c^ ± 0.4990
PCL/Gt-L2 bilayer	124.3^b^ ± 9.622	17.98^a^ ± 4.194	159.2^a^ ± 5.067	22.06 ^b^ ± 2.377	156.1^a^ ± 22.22	24.48^b^ ± 6.832

Different lower case letters within each column indicate significant difference (*p* < 0.05)

Degradation tests showed that the PCL scaffold had the significantly lowest weight loss percentage amongst all scaffolds throughout the immersion. The PCL/Gt-L2 bilayer scaffold showed a steady degradation rate from day 2 to day 14. Moreover, the PCL-L2 bilayer scaffold showed significant weight gain between days 2 and 7 days of immersion (*p* < 0.0001), after which it showed significant weight loss between 7 and 14 days (*p* = 0.0195). Furthermore, the PCL/Gt and PCL/Gt/BG scaffolds followed the same trend throughout the entire duration, showing an increase in weight loss from 2 to 7 days by approximately twofold (*p* < 0.0001) losing 33% of their weight after 7 days of immersion.

### Mechanical testing

**Fig. 9:**
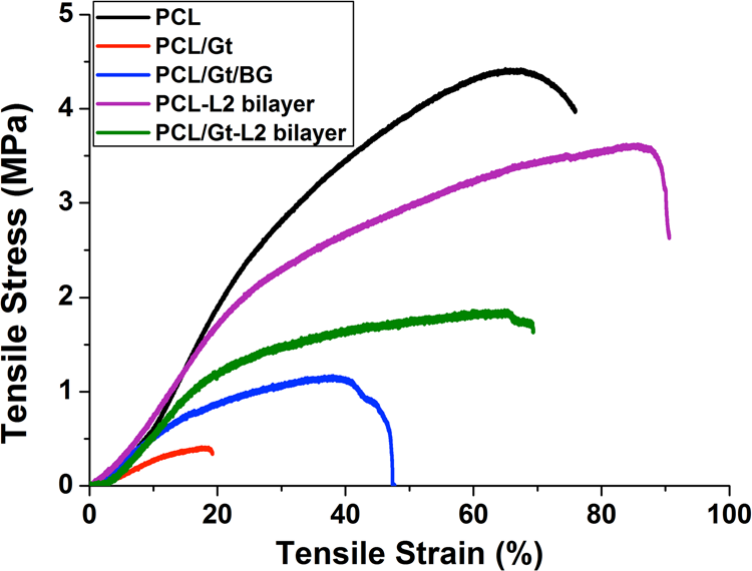
Stress-strain curves of the representative samples for each of the prepared scaffolds

The mechanical properties of the prepared scaffolds were assessed by comparing the tensile strength, elastic moduli and percentage elongation. As can be seen in Table 4, the PCL scaffold recorded significantly the highest mean tensile strength, followed by the PCL-L2 bilayer, PCL/Gt-L2 bilayer, PCL/Gt/BG composite scaffold and finally PCL/Gt scaffold, which showed the lowest tensile strength. The tensile strength of the PCL/Gt-L2 bilayer scaffold was fourfold higher than that of the PCL/Gt scaffold (*p* = 0.0093), while the difference between the PCL/Gt scaffold and the PCL/Gt/BG composite scaffold was insignificant (*p* = 0.1605). The prepared scaffolds showed the same trend for the elastic moduli measurements. The addition of Gt to PCL reduced the elastic modulus by ~80%. On one hand, the addition of BG nanoparticles in a composite single layer scaffold PCL/Gt/BG significantly improved the stiffness of PCL/Gt by ~2.2 times (*p* = 0.0356), while on the other hand, the addition of BG nanoparticles in a bilayer scaffold (PCL/Gt-L2) improved the stiffness by ~2.4 times (*p* = 0.0077). The stress-strain curves of representative samples from each scaffold are shown in Fig. 9. On comparing the elongation percentage of these curves, it was revealed that the addition of BG nanoparticles in the composite scaffold PCL/Gt/BG led to an increase in ductility by 21% in comparison to that of the PCL/Gt scaffold. On the other hand, there was a 49% increase in ductility between the PCL/Gt-L2 bilayer and PCL/Gt scaffolds.

**Table 4:** Mean and standard deviations (SD) values for tensile strengths and elastic moduli of the prepared scaffolds

Scaffold	Tensile strength (MPa)	Elastic modulus (MPa)
	Mean ± SD	Mean ± SD
PCL	4.25 ± 0.744^a^	13.8 ± 2.35^a^
PCL/Gt	0.404 ± 0.0826^d^	2.82 ± 0.490^c^
PCL/Gt/BG	1.20 ± 0.230^cd^	6.13 ± 0.422^b^
PCL-L2 bilayer	3.22 ± 0.707^b^	7.54 ± 1.90^b^
PCL/Gt-L2 bilayer	1.66 ± 0.530^c^	6.88 ± 2.07^b^

Different lower case letters within each column indicate significant difference (*p* < 0.05)

## Discussion

**Fig. 10:**
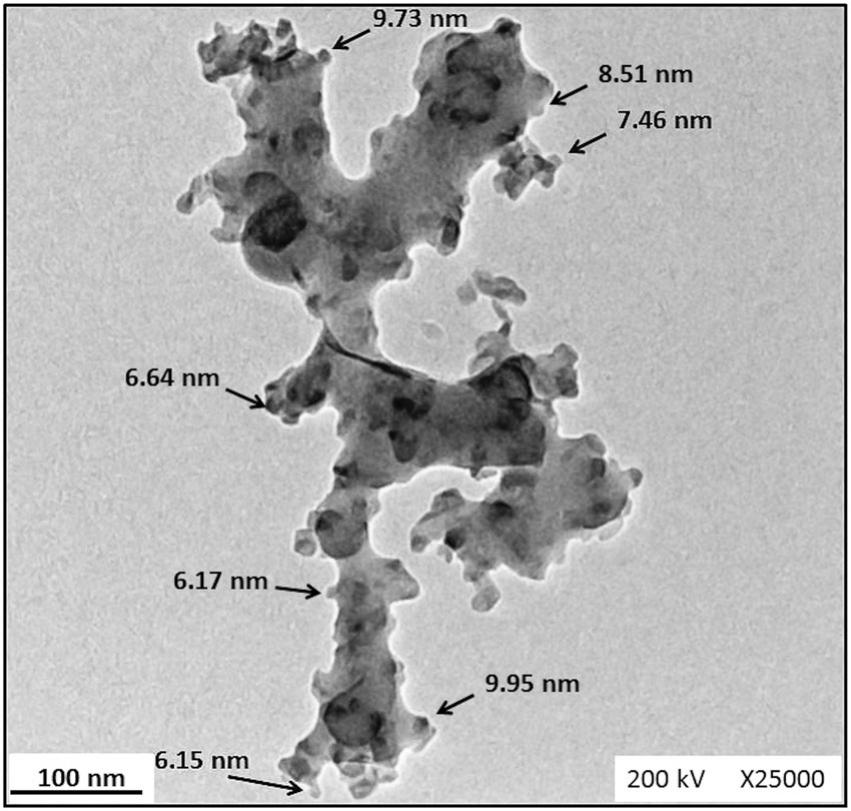
TEM image of BG nanoparticles at magnification ×25000. The sample was dispersed in ethanol at a ratio of 0.1 mg/mL followed by sonication for 30 min. Arrows indicate nanoparticles within the range of 6–10 nm in size

Nanostructured scaffolds improve osteoinduction and osteointegration significantly, since osteogenic cells identify nanoscale minerals and proteins, enhancing cellular interaction and functions [33, 34]. Accordingly, electrospinning was chosen in this study to fabricate fibers of diameters ranging in the nanoscale. The mean fiber diameter observed for PCL nanofibers was found to be in agreement with Ekram et al. [35]. However, the addition of Gt to PCL revealed a reduction in fiber diameter which was consistent with Lim et al. [36]. This was likely due to a higher viscosity of the PCL/Gt solution in comparison to that of PCL, which in turn required higher voltages to form a Taylor cone during electrospinning, thus reducing its fiber diameter [21]. BG incorporation increases the viscosity of the solution as well as its conductivity [22]. When the effect of BG on viscosity was greater than that of conductivity, the average fiber diameter increased as shown by the PCL/Gt/BG composite nanofibers. However, below a certain BG concentration, observed fiber diameters decreased with the addition of BG nanoparticles, as their effect on conductivity surpassed that of viscosity [22, 37]. The incorporation of BG nanoparticles induced a rough surface morphology, which was also reported by Lima et al. [37]. This surface roughness is preferable for cellular adhesion and proliferation [38]. As can be seen in Fig. 10, the TEM image of BG nanoparticles revealed spherical nanoparticles in the range of 6–10 nm in size. This confirms that the nanostructures observed in the TEM images of the BG incorporated nanofibers (PCL/BG and PCL/Gt/BG) correspond to BG nanoparticles and their aggregates.

The appearance of ultrathin fibers of diameters ranging between 15 and 30 nm forming a web-like structure is termed “nanonets” [39]. In this study, nanonets appeared in all scaffolds which was likely due to the high conductivity of formic acid, owing to its high dielectric constant (ε = 57.5). Moreover, BG increased solution conductivity, producing fibers of smaller diameter, which explains the increase in the distribution of nanonets in BG-containing scaffolds [37]. Similar nanonets were previously noted, as it was reported that their presence increased the scaffold’s mechanical properties, surface area and enhanced pore interconnectivity [40, 41].

**Fig. 11:**
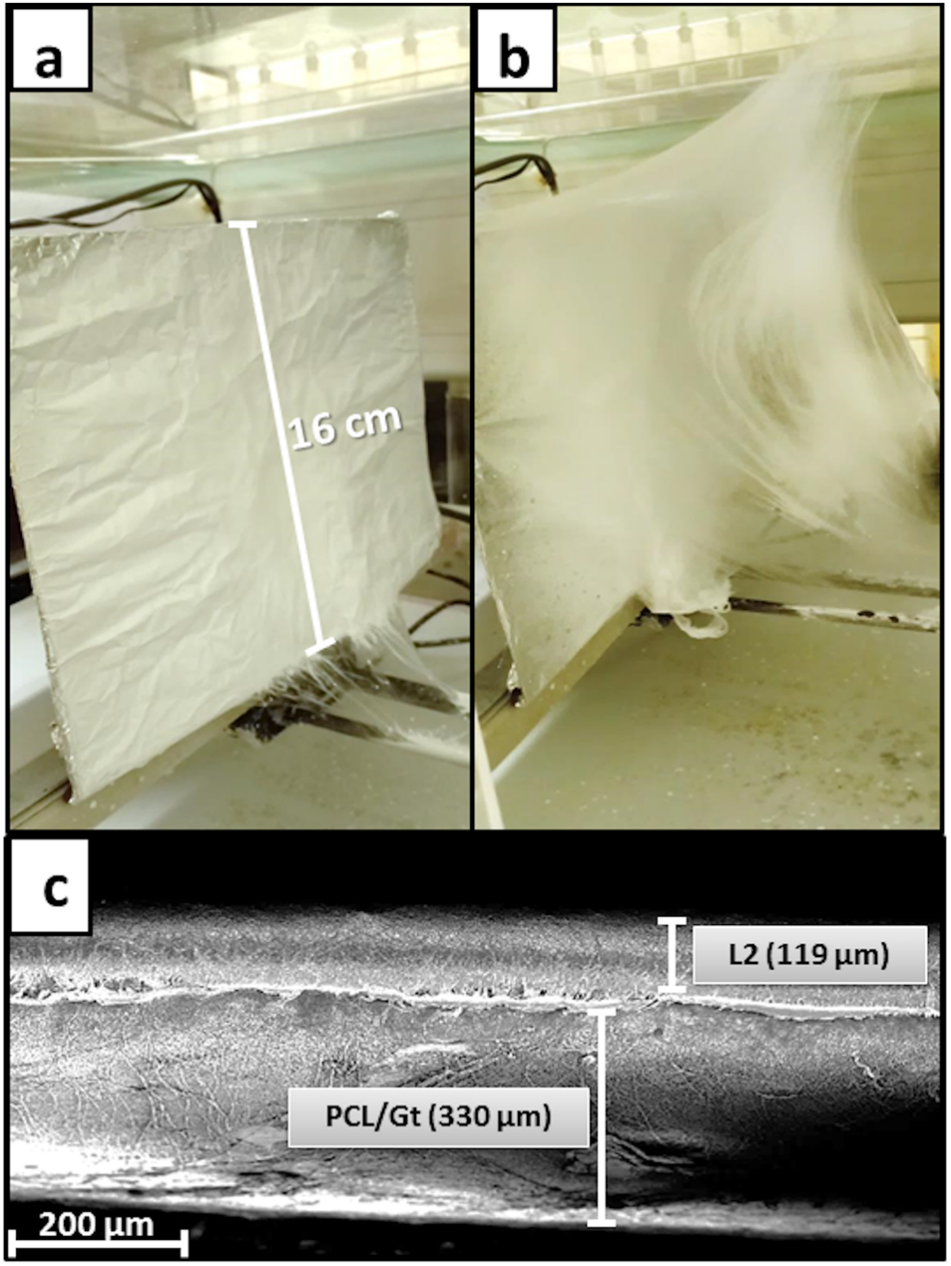
Fibers deposited on the aluminum foil covering the copper plate collector during a 6 h collection period of (**a**) PCL fibers and (**b**) PCL/Gt fibers. Cross sectional SEM image of (**c**) PCL/Gt-L2 bilayer. Scale bars indicate the thickness of both layers

During the fiber collection process, the fibers produced from the PCL/Gt solution exhibited a cotton wool appearance by time (Fig. 11b), which not only led to the highest average pore diameter amongst the fibers, but also led to an increase in fiber diameter during the fiber collection process. This could explain the reduction in surface area of the PCL/Gt scaffold measured using the N_2_ adsorption method as compared to pure PCL fibers (Fig.11a). Putti et al. [42] reported that morphological changes in the microstructure of fibers electrospun in air may be affected by the hydrophilicity of the polymer. Htike et al. [43] also observed an increase in the instability of the polymer jet at relative humidity between 50 and 70% in polyvinyl alcohol/soluble egg shell membrane (S-ESM) which contains natural collagen. They correlated this instability to the moisture absorbed by the (S-ESM) component. Although solutions of equal volumes were used to electrospin both layers of the PCL/Gt-L2 bilayer, there was a difference in their thickness as can be seen in the cross-sectional SEM image shown in Fig. 11c. The PCL/Gt layer was ~2.8 times thicker than L2 which could be attributed to the higher flow rate and voltage used for electrospinning the PCL/Gt solution as well as to the cotton wool structure formed.

As reported, high surface area of scaffolds would be more favorable as it would simulate the natural ECM and enhance cell adhesion and proliferation [22]. BET results showed that the PCL/Gt-L2 bilayer scaffold had higher surface area than the composite single layer scaffold PCL/Gt/BG, which was attributed to the difference in fiber diameters and total pore volume measured. Since the N_2_ adsorption method only measures porosities less than 300 nm in size, mercury intrusion porosimetry was used to measure scaffold porosity at the larger scales. This difference in scales accounts for the discrepancies between both methods in measuring the total pore volume. In addition, the adsorption-desorption isotherm measured by the N_2_ adsorption method revealed that all scaffolds exhibited a type II isotherm with H3 type hysteresis loop, which is indicative of a macroporous sample with pore sizes >50 nm [44]. In particular, H3 hysteresis was extremely narrow and was associated with capillary condensation within the mesopores and also indicated the presence of slit-like pores [31]. Mercury intrusion porosimetry showed the distribution of pores at a wider scale and revealed the presence of pore diameters up to 50–100 µm, this allows for osteoblasts which are 10–50 µm in size to penetrate within these pores. Moreover, the presence of a wide range of porosities presents an advantage as it allows the scaffold to maintain its elastic state after deformation as a result of mechanical stresses [45].

Furthermore, EDX analysis showed evidence of small traces of silica and calcium elements in the BG-containing scaffolds before immersion in SBF, which indicated the incorporation of BG. Their peaks were negligible in comparison to the main polymer elements (carbon and oxygen) owing to the very low concentration of BG used. In vitro bioactivity results showed that the PCL/Gt/BG composite scaffold contained very little phosphorus and calcium after 14 days of immersion, as revealed by EDX analysis, which was in agreement with Gonen, et al. [2], who reported no apatite formation when using 2.5% BG. As for the PCL/Gt-L2 bilayer scaffold, the SEM image showed the appearance of the typical cauliflower shaped structures, which represented hydroxyapatite-like crystals [20]. The resulting Ca/P molar ratio was 1.4, which was in agreement with the non-stoichiometric hydroxyl carbonate apatite, where values in the vicinity of 1.67 allow for a strong and stable connection with the surrounding living tissue [46, 47]. This finding showed that the incorporation of BG in a bilayer configuration could achieve bioactivity using a lower concentration of BG than that needed in a composite single layer scaffold. This lower BG concentration is of importance, as it was reported that BG nanoparticle concentrations above a threshold would induce scaffold brittleness due to particle agglomeration [20, 24, 48]. Cytotoxicity test suggested that the PCL/Gt-L2 bilayer expressed good biocompatibility after 48 h of incubation. Moreover, in accordance with ISO 10993-5, the percentage of cell viability recorded was considered to be non-cytotoxic [49].

The ability of a scaffold to swell allows cellular infiltration, nutrient diffusion and the removal of waste products. It also aids it in filling the defected area allowing close proximity of the scaffold to the adjacent living tissue [50]. Gt-containing scaffolds showed high water uptake due to the presence of carboxylate, amide and carbonyl groups that form hydrogen bonds with water molecules [28]. Moreover, the mean size of pores increased in Gt-containing scaffolds. High water solubility and brittleness of Gt nanofibers could be enhanced by crosslinking using a number of methods. The most widely used method is chemical crosslinking; however, it has major drawbacks such as reducing the porosity of the scaffold due to changes in the morphology of the fibrous structure [51]. An alternative method used to enhance the mechanical properties and degradation of gelatin is blending it with a synthetic polymer such as PCL which is a hydrophobic polymer with very limited swelling capacity [11, 52]. After 2 days of immersion in SBF, the PCL/Gt and PCL/Gt/BG scaffolds reached their maximum swelling capacity, after which the fibers began to degrade via hydrolysis [28]. The presence of BG increased the swelling capacity of the scaffolds by allowing rapid exchange of alkali elements from the nanoparticles with protons from water molecules, resulting in bulk and surface changes in the nanofibers, and increased scaffold hydrophilicity [53].

Moreover, the weight loss shown for the pure PCL scaffold was in agreement with Kouhi, et al. [48]. The PCL-L2 bilayer scaffold showed significant weight gain after 7 days of immersion, which could be due to amorphous calcium phosphate deposition, which led to a net gain in weight. This was followed by progressive scission of the polymeric chains due to the increase in water uptake by the scaffold [37]. PCL/Gt-L2 bilayer scaffold showed a steady rate of degradation between 2 and 14 days, while the PCL/Gt/BG composite scaffold reached its maximum weight loss at 7 days.

Tensile strength results showed that the PCL scaffold had the highest strength among all scaffolds, which is higher than that reported by EL-Fiqi et al. [24] and Kouhi et al. [48]. Conversely, the PCL/Gt scaffold showed the lowest tensile strength among the tested scaffolds. In general, PCL nanofibers exhibit a toughening mechanism; in particular, after a crack forms, the fibers align ahead of the crack tip and transverse to the crack propagation direction, inducing ductile fracture. In contrast, Gt-containing fibers exhibit brittle fracture due to the random network morphology ahead of the crack tip [54, 55]. Its low tensile strength could also be explained by the weak physical interactions between the different polymers, which cause micro-phase separation and easier slippage of chains under mechanical loading [11]. Moreover, osteoblast proliferation is enhanced by the stiffness of the biomaterial [45]. The addition of BG nanoparticles in the composite single layer scaffold (PCL/Gt/BG) improved tensile strength, elastic modulus and ductility in comparison to those of the PCL/Gt scaffold. Nevertheless, incorporating BG as a surface layer in the bilayer scaffold PCL/Gt-L2 had positive effects on the same properties, but with a much higher significance. This was in agreement with El-Fiqi et al. [24], who reported an increase in the elastic moduli with the addition of mesoporous BG nanoparticles. As BG nanoparticles act as fillers, they reinforce the matrix by forming secondary bonds with the polymer [56]. Moreover, their presence led to an increase in ductility, likely in relation to the yielding phenomenon caused by BG nanoparticle debonding from the polymer matrix [57]. On the contrary, the PCL-L2 bilayer had lower tensile strength and elastic modulus in comparison to the PCL scaffold. This could be due to the increase in inhomogeneity of the fiber diameters [20].

Based on the results obtained in this study, it could be highlighted that fabricating a bilayer scaffold using electrospinning and evaluating its properties are of importance in the field of BTE and could provide enhanced biological, physical and mechanical properties as opposed to their single layer counterparts. Further in-vivo studies are recommended to assess the scaffold’s performance clinically.

## Conclusion

The incorporation of PCL with Gt and BG into an electrospun bilayer scaffold in comparison to a composite single layer scaffold PCL/Gt/BG enhanced surface area, increased total pore volume, provided sufficient bioactivity by forming a hydroxyapatite-like layer which covered the entire surface of the scaffold after 14 days of immersion in SBF, and maintained a steady degradation rate. Moreover, PCL/Gt-L2 bilayer exhibited a significantly enhanced tensile strength with respect to the PCL/Gt scaffold, as well as elastic modulus and ductility. Furthermore, the bilayer showed good biocompatibility and was considered non-cytotoxic. Hence, the PCL/Gt-L2 nanofibrous bilayer scaffold demonstrated its potential to be used in the field of bone tissue engineering.
